# Toward Sustainable Aesthetic Transparent Wood from a Fast-Growing Hardwood Species: Paulownia Wood Templates Infused with Epoxy Bioresin

**DOI:** 10.1021/acsomega.5c09133

**Published:** 2026-01-08

**Authors:** Francesco Bolognesi, Emanuele Galvanetto, Leonardo Duranti, Andrea Bianco, Marco Togni, Alessandra Bianco

**Affiliations:** † Dipartimento Ingegneria dell’Impresa “Mario Lucertini”, Consortium INSTM RU “Roma Tor Vergata”, 9318 Università degli Studi di Roma “Tor Vergata”, Via del Politecnico, 00133 Roma, Italy; ‡ Dipartimento di Ingegneria Industriale (DIEF), 9300 Università degli Studi di Firenze, Via di Santa Marta 3, 50139 Firenze, Italy; § Dipartimento di Scienze e Tecnologie Chimiche, Consortium INSTM RU “Roma Tor Vergata”, Università degli Studi di Roma “Tor Vergata”, Via della Ricerca Scientifica, 00133 Roma, Italy; ∥ INAF Osservatorio Astronomico di Brera, via Bianchi 46, 23807 Merate, Italy; ⊥ Dipartimento di Scienze e Tecnologie Agrarie, Alimentari, Ambientali e Forestali (DAGRI), Università degli Studi di Firenze, Piazzale delle Cascine 18, 50144 Firenze, Italy

## Abstract

Lignocellulosic biomass is an attractive renewable resource for the development of engineered materials in the framework of a green economy. Transparent wood (TW) products show great potential in green architecture, energy saving building, optical devices, electronics, energy storage, and conversion devices. The fabrication of TW products proceeds through the delignification of bulk wood samples followed by infiltration with a refractive index-matched polymer. This study is focused on *Paulownia tomentosa* (Thunb.) Steud., a fast-growing species rarely investigated, is characterized by low-density wood and a distinct early to late wood pattern in each growth ring. Delignification was performed by a conventional bleaching route. Aesthetic wood was obtained by infusing the delignified templates with an epoxy bioresin. The characterization was performed by nondestructive techniques: optical microscopy, scanning electron microscopy, X-ray diffraction, FT-IR spectroscopy, Raman spectroscopy, and UV–vis spectroscopy. Thermal degradation profiles were acquired by thermogravimetry, and mechanical strength was evaluated by tensile tests. The chemical treatment led to 10–15% dry mass loss, mainly due to the removal of lignin, and the efficacy of delignification was comparable for transversal and longitudinal bulk wood. The removal of a minor amount of hemicellulose, especially for axial samples, also occurred. Delignified templates preserved dimensional stability in wet and dry states and showed increased Segal crystallinity index (CI), reduced thermal stability, improved total optical transmittance, increased brightness, and loss of tensile strength. The infusion with the bioresin led to aesthetic wood characterized by increased optical transmittance (up to 60% at 800 nm) combined with fully recovered tensile strength and preserved natural wood features clearly visible to the naked eye.

## Graphical abstract

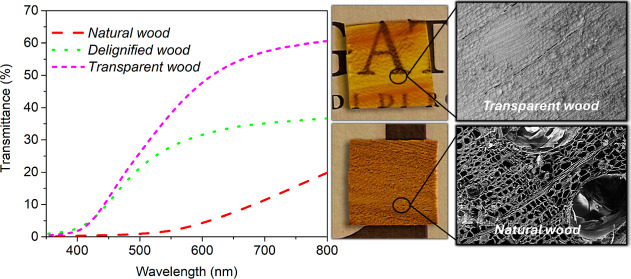

## 1 Introduction

Materials sustainability is a broad multidisciplinary research area promoting the industrial growth within the complex paradigm of the green economy.^1,2^ In this context, wood has received great attention as a versatile, inexpensive, recyclable, and biodegradable material.^3–5^ Wood is composed of cellulose (∼40–47% by weight), hemicellulose (∼25–35% by weight), lignin (∼16–31% by weight), and a small amount of low molecular weight substances.^6–9^ Cellulose fibrils are embedded in a matrix composed of lignin and hemicellulose giving rise to a hierarchical architecture in which the anatomical elements (i.e., tracheids in softwoods, vessels, and fiber cells in hardwoods) feature a multilayer wall-around-lumen structure made up of an outer primary layer (P layer), an inner multiple secondary layer (S_1_, S_2_, and S_3_ layer), and the middle lamella (ML) located at cell corners and between adjacent cells. The properties of the cell wall are mainly controlled by the 5–10 μm thick S_2_ layer that comprises the majority (70–90%) of the cell volume.^7,9,10^ Hemicelluloses are amorphous short chain branched heteropolysaccharides tightly bonded, but noncovalently to the surface of cellulose microfibrils. Hemicelluloses are made up of hexose (glucose, mannose, and galactose) and pentose (xylose and arabinose) units. In addition, the hemicellulose structure contains uronic acids (d-glucuronic acid, d-galacturonic acid, and 4-O-methyl-d-glucuronic acid) and acetyl groups. The composition of hemicellulose varies and is dependent on the plant source. In softwoods, glucomannans are the most common type of hemicellulose, whereas hardwoods contain mostly xylans. The backbone of xylan comprises β1–4-linked xylose. Xylan is commonly decorated with arabino furanose or glucuronic acid side chains, with the degree and type of decoration depending on the source of the xylan.^6,7,10,11^

Lignin is an amorphous cross-linked 3D macromolecule based on phenylpropane units, mainly concentrated in the ML region. In hardwoods, the prevalent building blocks of lignin are syringyl (S) and guaiacyl (G) units, derived from sinapyl and coniferyl alcohol, respectively. In softwoods, lignin mostly contains guaiacyl (G) units combined with a small amount of *p*-hydroxyphenyl (H) units derived from *p*-coumaryl alcohol. The phenyl propane units are bonded by a series of characteristics such as β-*O*-4, α-*O*-4, β-5, and β–β linkages.^9,12–14^

Such a chemical nature is responsible for the peculiar chemical and physical properties of lignin. For example, the low solubility is due to intermolecular hydrogen bonding and π–π stacking, whereas the excellent UV adsorbing (200–400 nm) and antioxidant properties are mainly ruled by the aromatic character of the phenylpropane units and by several chromophore structural elements whose mixture determines also the color of lignin due to their absorbance in the visible light range (400–750 nm).^14^

In the past decade, the development of transparent wood (TW) has represented a leading-edge topic since it has shown great potential in emerging fields, including energy-saving building, optical devices, electronics, energy storage, and conversion.^4,15–17^

The fabrication of TW first requires the delignification of bulk wood samples, followed by infiltration of the resulting template with a refractive index-matched polymer.^18–21^

Delignification techniques are powerful chemical and physical strategies aimed to modify and/or remove lignin from wood in order to alter its features at multiple scale lengths.^9,21^ Chemical delignification routes, based either on pulping (i.e., nucleophilic reactions in alkaline Na_2_SO_3_ or Na_2_S solutions) and/or on bleaching (i.e., electrophilic, radical, or oxidative reactions in H_2_O_2_, ClO_2_, NaClO, or NaClO_2_ solutions), allow the deconstruction and fragmentation of lignin and promote hydrophilicity, producing cellulose-rich templates (delignified wood) characterized by the hierarchical architecture typical of the pristine material associated with tailored microstructural and physical properties.^4,9,21,22^

The selection of polymers for the final infiltration stage is mainly driven by two features, high transparency and refractive index matching with wood minimizing refraction and scattering of light at the interface between the media. Currently, the fabrication of TW mainly relies on thermosetting epoxy resins and poly­(methyl methacrylate) (PMMA). Other thermoplastic polymers such as poly­(vinyl alcohol) (PVA), polyvinylpyrrolidone (PVP), *n*-butyl methacrylate, polystyrene (PS), and dibutyl phthalate have been rarely investigated.^20,21,23^

Nowadays, most of the materials research is driven by the concepts of sustainable development in terms of environmental protection and reduction of energy consumption.^24^ In this perspective, based on the inspiration of the frontier topic of TW and in view of the increasing demand for novel functional material for home decoration, translucent wood-based materials (aesthetic wood) are considered an excellent resource for green architecture, design and green building.^25–27^ The development of aesthetic wood is based on the idea of spatial-selective removal of lignin from early wood (EW) regions to improve the optical transmittance and simultaneously preserve the natural pattern of the timber.^25^ Aesthetic woods are considered multifunctional materials showing optical transparency, UV-blocking, thermal insulation, scalability, and aesthetics.^25^

Presently, the successful translation to the market of these biosourced composites depends on the performance in the outdoor environment, in terms of UV resistance, biological resistance, and self-cleaning^24^ and on the evaluation of the environmental footprint.^20,28,29^

Regarding environmental degradation, Wachter et al.^24^ reported that, compared to natural wood (NW), acrylic-based TW shows higher resistance to wood-decay fungi and higher resistance to ignition combined with lower carbon monoxide yield. On the other side, they showed higher values of heat release rate and suffered higher sensitivity to photodegradation processes, resulting in the decay of transmittance and lightness.

Regarding environmental sustainability, Rai et al.^20^ performed life cycle analysis (LCA) for TW production at different scale levels. The lowest environmental impact was obtained for TW products delignified by sodium sulfite-based strategies^22^ followed by infiltration of wood templates with epoxy resins. The same authors also calculated the potential environmental impact of the products after the estimated shelf life by end of life (EOL) analysis.^20^ In the EOL phase, TW resulted more harmful than glass due to the release of chemicals associated with the decomposition in the environment, especially for the incineration scenario. More recently, Wu et al.^28^ quantified the environmental footprint from production and disposal of TW in terms, among others, of global warming, air pollution, ecotoxicity, natural resource depletion, habitat alteration, and water depletion. This LCA study demonstrated that TW obtained by modified lignin bleaching method, epoxy infiltration, and cellulose volume fraction up to 65%, resulted in a 57% reduction in global warming impact compared to common transparent petroleum-derived thermoplastic polymers such as poly­(methyl methacrylate) (PMMA) and polycarbonate (PC).

Therefore, future directions for sustainable large-scale production of TW urgently demand switching to index-matching biodegradable polymers or to biologically derived polymers.^28,29^

This study is focused on *Paulownia tomentosa* (Thunb.) Steud. (Paulownia) also called Royal Paulownia, Empress Tree, or more commonly, Princess Tree. Paulownia is a fast-growing hardwood native to China, widely cultivated in wood arboriculture plants, including Central-Northern Italy.^30–32^ In spite of the large employment of paulownia for conventional uses and, due to the high cellulose content, i.e., 46 wt %, Kürschner cellulose, 24 wt % pentosans and 20 wt %, Klason lignin,^7^ recent application as lignocellulosic biomass to produce bioethanol and biohydrogen,^33^ the potentiality of this low-density wood is still underestimated in the field of TW products.^34,35^ On such a basis, the idea is to perform a proof-of-concept study to evaluate Paulownia for the fabrication of sustainable aesthetic TWs from a hardwood species. In fact, for such a purpose, softwoods (Douglas fir, Chinese fir, and New Zealand pine) are nearly exclusively investigated due to their regular ordered organization of tracheids within the growth ring of EW and late wood (LW) that favors the achievement of a sharp aesthetic result.^25,26,36^ Moreover, it is worth mentioning that fast-growing *Ochroma pyramidale* (Cav. ex Lam.) Urb. (Balsa), the outermost employed hardwood species for the realization of TW products,^15,16,20,37,38^ is not suitable for such a purpose due to the typical diffused porosity.^15,25^ It is worth reminding that very thin wood templates derived from the delignification of hardwood species might present signs of detachments, especially along the wood grain.^22^

A time-effective in situ delignification process based on sodium chlorite solution has been selected.^25^

It is worth clarifying that, despite the recently ascertained higher environmental impact compared to other chemical treatments,^20,28^ most of the TW products are obtained through chlorite-based delignification routes.^22^ On such a basis, the rationale under this study was to validate the bioderived ER using wood templates delignified by this mainstream treatment. To preserve as much as possible the aesthetic appeal of wood and, at the same time, reduce the environmental impact of the chemical procedure mostly associated with the length of the treatment,^20,28^ the minimum time of exposure was chosen.^29^

TW products were obtained by infusing under vacuum the delignified bulk wood samples with an ultralow viscosity bioresin derived from plant sources not competitive with food sources or food-based agriculture. Presently, to the best of our knowledge, this kind of thermoset polymer has not yet been considered for the fabrication of aesthetic TWs.

Samples were characterized by the following nondestructive techniques:^39^ optical microscopy (OM), scanning electron microscopy (SEM), infrared spectroscopy (ATR-FTIR), Raman spectroscopy, X-ray diffraction analysis (XRD), color spectrometry, and total optical transmittance in the UV–vis range. Moreover, the thermal degradation profiles were acquired by thermogravimetry (TG), and the mechanical properties were determined by uniaxial tensile tests.

## 2 Experimental Section

### 2.1 Preparation of Bulk Wood Samples

Paulownia (*Paulownia tomentosa* (Thunb.) Steud.) transversal cut (T-wood) size 20 × 20 × 1 mm, 20 × 20 × 3 mm wood samples were prepared using a chop saw (FC 350 RAP, OMS, Italy). For the sake of comparison, longitudinal cut (L-wood) 20 mm × 40 mm × 1 mm samples were also considered.

### 2.2 Delignification Procedure

The bleaching aqueous solution was prepared by dissolving 1% wt of NaClO_2_ in the acetate buffer solution (pH ∼ 4.6).^25^ Samples were soaked in the delignification solution at the boiling temperature (about 105 °C) without stirring for 3 h, washed several times with cold distilled water until a colorless solution was obtained, and finally stored in ethanol.^25^ In the following, the obtained bulk wood templates and pristine wood samples will be designed, respectively, as delignified wood (DW) and NW. Quantitative evaluation of dry mass loss was determined on a set of four specimens (T-wood 20 × 20 × 3 mm and L-wood 20 × 40 × 1 mm) using an analytical balance. DWs were previously washed three times alternatively with ethanol and acetone and then oven-dried at 105 °C for 24 h.^40^

### 2.3 Bioresin Infusion

The selected bioresin is a conventional epoxy thermoset polymer whose chemicals are derived from plant-based sources. The key component epichlorohydrin is manufactured using renewable plant-based and glycerol is employed in place of petroleum-based propylene. Additionally, the raw materials are coproducts or waste products of industrial processes and thus do not compete with food sources or food-based agriculture. The overall plant-derived content is approximately 31%, which is among the highest for epoxy resins devoted to large-scale applications (https://www.easycomposites.co.uk/). DW samples have been previously dried overnight to remove residual ethanol and then infused with the previously mentioned low viscosity bicomponent epoxy bioresin (i.e., viscosity at 20 °C: resin, 1350 mPa·s; hardener, 7 mPa·s; combined, 185 mPa·s) (IB2, Easy Composites, Park Hall Business Village, United Kingdom). Before the intrusion, the resin was degassed in a vacuum dryer through a rotary vane pump. After intrusion, the specimens were placed between two glass plates for the curing treatment and cured for 24 h at room temperature. Then, a postcuring treatment at 80 °C for 8 h was performed according to the technical data sheet. In the following, the resulting samples will be designed as TW. The weight gain percentage was performed using an analytical balance; at least five specimens have been considered. For the sake of comparison, natural T-wood and L-wood have been analogously treated to obtain a couple of control samples (CS). A neat bioresin sample processed and cured in the same conditions was also prepared. According to the producer, the following mechanical properties are expected: tensile modulus 2.64 GPa, tensile strength 60 MPa, elongation at break 9.5%, flexural modulus 2.61 GPa, flexural strength 101 MPa, compressive strength 82 MPa, and impact resistance 89 kJ/m^2^ (https://www.easycomposites.co.uk/). The transmittance of the pale amber hardened bioresin increased continuously from 65 to 90% within 300 and 450 nm and then remained constant until 800 nm (Figure S1, Supporting Information). According to the database (https://www.easycomposites.eu/), the refraction index of the epoxy infusion bioresin (a clear liquid) and the hardener (an amber liquid) are 1.549 and 1.498, respectively. The values are fully in line with conventional epoxy resins.^18,19,23^

### 2.4 Characterization Techniques

- Dry density and moisture content were determined on a set of four specimens following a procedure adapted from EN 13183-1:2002, moisture content of a piece of Sawn timber—Part 1: determination by the oven dry method. For density, the reference standard was ISO 13061-2:2017, physical and mechanical properties of wood—test methods for small clear wood Specimens—Part 2: determination of density.
- OM observations were performed on natural and delignified wood samples derived from samples finely cut by microtome (SM2010R, Leica, Wetzlar, Germany). The surface morphology was observed adopting an optical stereo microscope (S9D, Leica, Wetzlar, Germany). Photos have been taken using a 12MP camera (Flexacam C1, Leica, Wetzlar, Germany) mounted on the microscope.
- SEM: delignified woods (DW) derived from microtome-cut wood samples (SM2010R, Leica, Wetzlar, Germany) were dried in an oven for 24 h at 105 °C, gold-sputtered (EMITECH K550X sputter coater, Quorum Technologies Ltd., UK), and then observed using a field emission scanning electron microscope (Leo-SUPRA 35, Carl Zeiss SMT, Oberkochen, Germany) operating at a low-range accelerating voltage (5 kV). The microstructure of gold-sputtered (Q150R ES; Quorum technologies Ltd., UK) TWs and CSs was investigated by a FE-SEM (EVO 40, Zeiss, Oberkochen, Germany) operating at an accelerating voltage of 8 kV. Prior to the observation, samples were smoothly polished with P1000 sandpaper.
- Infrared spectroscopy (FT-IR) was performed on microtome-cut wood samples (SM2010R, Leica, Wetzlar, Germany) using a Fourier transform infrared spectrometer (FTIR-4600, Jasco Inc., Tokyo, Japan) rigged with the attenuated total reflection (ATR) accessory (ATR PRO ONE X, ZnSe crystal). DW has been previously dried overnight at room temperature. Spectra were collected through 128 scans at a resolution of 4 cm^–1^ in 500–4000 cm^–1^ spectral range. Background was collected before the measurements.
  To perform a semiquantitative analysis assessing the effect of the delignification treatment on the main wood components, the ratios between the areas of the FT IR bands at 1734 cm^–1^ (reference for hemicelluloses), 1506 cm^–1^ (reference for lignin), and 1159 cm^–1^ (reference for holocellulose) were calculated using a single linear baseline for each peak of interest.^22,41–43^ All the spectra were processed using Spectragryph 1.2 software.
- Raman spectroscopy was performed on delignified samples derived from a microtome-cut wood specimen (SM2010R, Leica, Wetzlar, Germany) previously dried overnight at room temperature. The instrument is composed of a BWTEK Exemplar Pro coupled with a fiber-fed Cleanlaze laser module (785 nm, 300 mW, spot size about 100 μm) operating in macro mode. The spectrum was processed using Spectragryph 1.2 software, subtracting an adaptive baseline (15% coarseness).
- Thermogravimetry analyses (TGA) were performed on 10 mg of powdered samples placed in an open alumina crucible under 100 mL/min nitrogen flow using a TG-DSC 1 (STAR system, Mettler Toledo, Columbus, United States). The chosen thermal treatment includes a first isothermal step at 115 °C for 10 min to remove the hygroscopic water, followed by a 20 °C/min ramp from room temperature to 900 °C.^44^
- XRD: the diffractograms of powdered samples were collected using a diffractometer (X-Pert Pro, Philips, Amsterdam, The Netherlands) with weighted Cu Kα1 and Kα2 average radiation λ = 1.5418 Å in the range 2θ = 5°–55° with 0.01 step size and 1 s time per step. To estimate the crystalline fraction in the investigated samples, the Segal crystallinity index (CI) method was chosen. The Segal CI was calculated according to the following formula (eq 1)

1 $$Segal\;CI=100\times \left(I_{200}-I_{am}\right)/I_{200}$$

where *I*_200_ is the intensity of the (200) peak (2θ range 22°–23°), taken as representative of the total amount of crystalline and amorphous components, and *I*_am_ is the intensity of the minimum between the (110) and the (200) peaks (2θ about 18°) associated only to the overall content of the disordered domains.

- The Segal peak height, peak deconvolution, amorphous subtraction, and, more recently, Rietveld refinement are the most widely applied methods to interpret XRD results; it is worth mentioning that the Segal method might overestimate the CI with respect to the other methods.^45,46^ The cellulose crystallite/microfibril size τ, defined as the lateral crystallite dimension in the direction normal to the crystallographic plane, was determined from the FWHM of XRD peaks according to the Scherrer eq (eq 2)

2 $$\tau =\frac{k\lambda }{\beta \cos\;\theta }$$

where λ is the radiation wavelength used in the XRD experiment, β is the peak fwhm, θ is the Bragg angle, and *k* is a constant, depending on the crystallite aspect ratio and distribution, whose value is usually close to unity.

Since the reflection (200) is the most intense and resolved, it is commonly used for crystallite size evaluation.^47^According to Garvey et al.,^48^the (200) peak was fitted with Voigt functions, and, taking *k* = 1, the crystallite size was calculated.

- UV–vis spectroscopy was performed on NW, DW, TWs, CSs, and neat bioresin. Before the analysis, wood samples were cleaned on the surface using a microtome (SM2010R, Leica, Wetzlar, Germany). Measurements were performed using an integrating sphere mounted on an ultraviolet–visible spectrophotometer (V-760, Jasco, Tokyo, Japan) in the 350–800 nm range, with an acquisition step set at 2 nm. Delignified specimens have been previously dried overnight at room temperature.
- Surface color was investigated using a color spectrophotometer (YS3020, 3nh, Guangzhou, China). The observation was performed using an observation spot of 4 mm of diameter, with an observer angle of 2°, and D65 as illuminant. Bulk samples, previously finely cut by microtome (SM2010R, Leica, Wetzlar, Germany), were dried overnight. The color code was determined according to the CIELAB system, where *L* is brightness (0 dark, 100 bright), whereas *a* and *b* are the chromatic coordinates. In detail, *a* is the red/green degree (+red, −green) and *b* is the yellow/blue degree (+yellow, −blue). The greater the absolute *a* and *b* values, the deeper the color.^26,49,50^ Each sample was measured in at least two replicates, and the mean values of *L*, *a*, and *b* were calculated.
  The color change (Δ*E*) is evaluated using eq 3 ^49,50^
3 $$\Delta E={\left(\Delta L^{2}+\Delta a^{2}+\Delta b^{2}\right)}^{1/2}$$

- Tensile tests were carried out on rectangular wood-L samples (size around 40 mm, width 4–5 mm, and thickness 1 mm) using a universal testing machine (LRX, Lloyd, Worthing, United Kingdom) at a 2 mm/min strain rate, equipped with a 500 N load cell and screw side action grip.^37^ Tensile tests were performed on neat wood samples stored in room conditions (moisture content 7.6 ± 0.4%).^51^ Delignified specimens were previously dried overnight at room temperature. For each group of samples (i.e., NW, DW, and TWs), at least five specimens were tested. Data have been statistically analyzed using one-way analysis of variance to assess significant differences (*p* < 0.05) between different groups of samples.^52^

## 3 Results and Discussion

On the macroscale, wood-T sections of Paulownia show annual growth ring patterns developed in spring EW and in summer LW. Usually, EW is wider, weaker, and lighter in color. Paulownia is a cultivation fast-growing hardwood species characterized by a semi-ring porous structure with large vessels (∼100 μm) mainly located in the EW region.

The vessel diameter normally decreases from the earlywood to the latewood, making annual growth rings easy to see in cross-section (Figure 1). The calculated dry density is 0.29 ± 0.03 g/cm^3^.^33^ SEM micrograph in Figure 2a shows the typical porous honeycomb-like structure of hardwoods. The low density and loose wood structure constructed by large-diameter fiber cells (Figure 2a) are typical of fast-growing hardwoods. Such features make these species highly accessible to subtractive and additive chemical modifications.^9,24^

**Figure 1:**
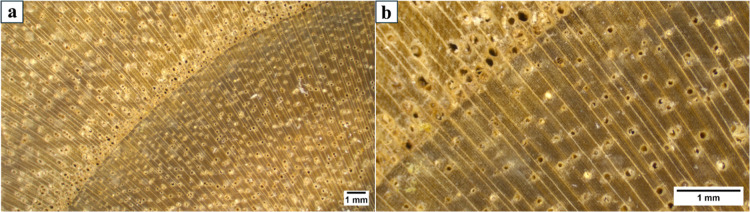
Optical microscopy images of transversal-cut (T) Paulownia wood from lower (a) to higher (b) magnification.

**Figure 2:**
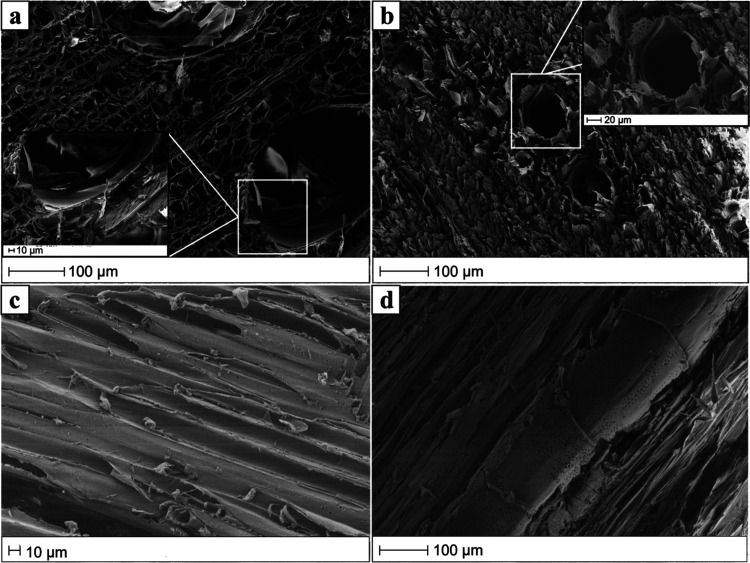
Scanning electron microscopy (SEM) micrographs of oven-dried samples: (a) natural T-wood, (b) delignified T-wood, (c) natural L-wood, and (d) delignified L-wood.

After 3 h of treatment, the dry mass loss was about 9.7 ± 0.5% and 14.6 ± 5.1%, respectively, for T-wood and L-wood. Interestingly, the chosen parameters of chemical delignification allowed to contain significantly the removal of wood tissue, which is usually much higher (typically around 30 wt %) and is responsible for challenging practical issues during the fabrication of TW products on a large scale.^53,54^

Figure 2 compares, respectively, the SEM micrographs of T-wood and L-wood of natural and delignified samples. Oven-dried delignified material showed remarkable contraction of vessels and fibers and loss of surface integrity. Interestingly, L-cut and T-cut DW samples preserved the shape and size of pristine wood after drying either in an oven or at room temperature (see Figure 7 in the following discussion).

Nondestructive techniques (FT-IR spectroscopy, Raman spectroscopy, and UV–vis spectroscopy) and thermogravimetry (TG) have been successfully combined for a comprehensive investigation of lignocellulosic biomass.^22,55,56^

The normalized ATR-FTIR spectra in the range 800–1800 cm^–1^ of pristine and delignified bulk wood samples are compared in Figure 3.^22,57^ The patterns of both T-cut and L-cut DW showed a sharp decrease of the fingerprint absorption bands of lignin aromatic moiety at 1593 cm^–1^ **(3)** and 1505 cm^–1^ **(4)**, assigned to vibration modes of the aromatic rings and at 1457 cm^–1^ **(5)** due to bending of C–H bonds of methylene (–CH_2_) and methyl groups (–CH_3_).

**Figure 3:**
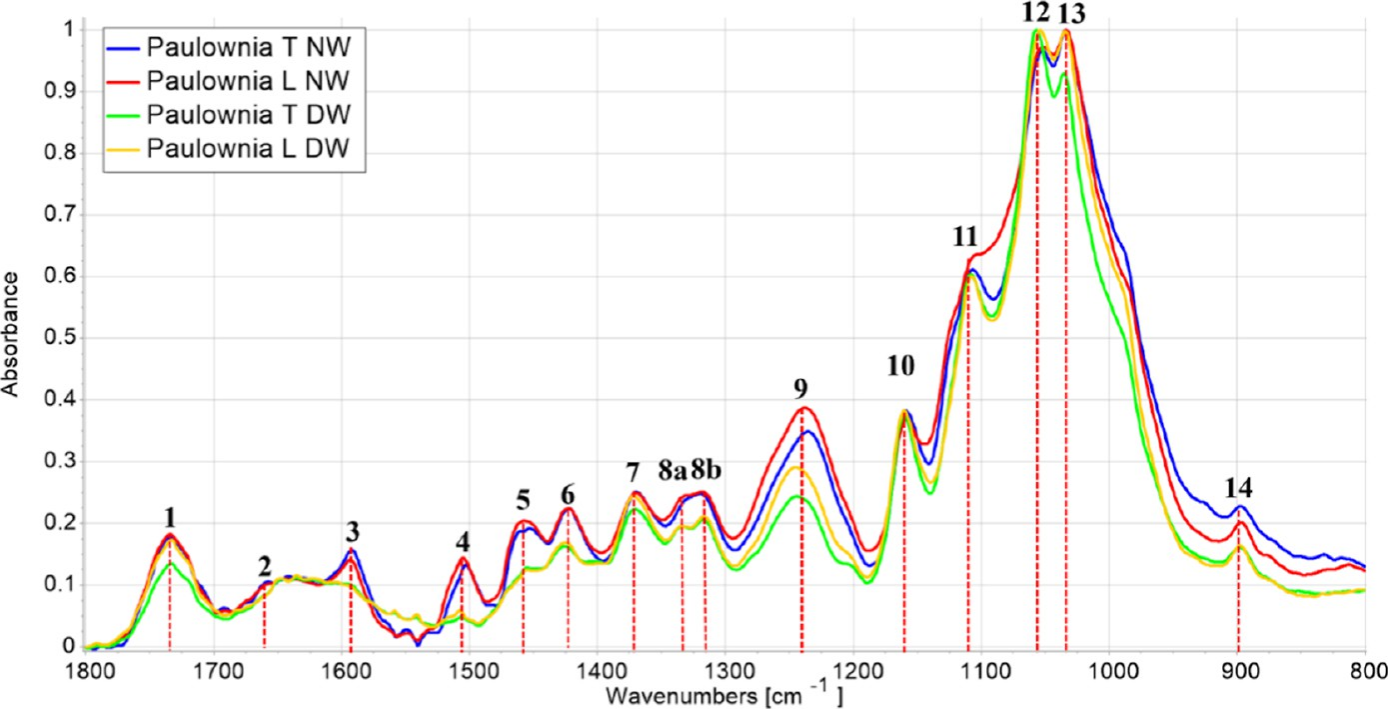
Normalized FT-IR spectra of natural woods (NW) and delignified woods (DW) for transversal (T) and longitudinal (L) cut. For visualization purposes, the individual spectra were normalized to the highest peak. Numerical annotations are explained in the text.

Accordingly, the weakening of peak **(9)** at 1236 cm^–1^, associated with the deformation of different chemical groups including the C–O stretching of syringyl methoxy groups (–OCH_3_) of lignin, has also been observed. Notably, the following peaks persisted: **(1)** 1734 cm^–1^ (medium) related to carbonyl groups (C=O) of xylan/glucomannan acetyl esters in hemicellulose, **(9)** 1236 cm^–1^ (medium) associated with the uronic acid groups of hemicellulose and lignin-hemicellulose ester bonds, **(10)** 1158 cm^–1^ (medium) due to asymmetric stretching of C–O–C in cellulose and hemicellulose, and **(14)** 900 cm^–1^ (small) associated with xylan vibration modes, a characteristic band of hardwood species.^42,43,49,58–60^ These results suggest that the chemical delignification process was effective to degrade the aromatic backbone of lignin, maintain the covalent bonds with hemicellulose, and preserve cellulose.

Further, the authors propose a semiquantitative method based on the analysis of FTIR spectra to relatively evaluate the effect of the chemical delignification treatment on the composition of Paulownia wood. Particularly, to relatively assess the loss of hemicellulose and lignin, the ratios of the area of the bands at 1734 cm^–1^, 1506 cm^–1^, and 1159 cm^–1^ were determined (paragraph 2.4). The results are reported in Table 1.

**Table 1:** Ratio between the Area of the FT-IR Peaks at 1734 cm^–1^ and 1159 cm^–1^ (*A*_1734_/*A*_1159_) Is Related to the Ratio between Hemicellulose and Holocellulose (Hem/Hol); the Ratio between the Area of the FT-IR Peaks at 1506 cm^–1^ and 1159 cm^–1^ (*A*_1734_/*A*_1159_) Is Related to the Ratio between Lignin and Holocellulose (Lig/Hol)^a^

sample	*A*_1734_/*A*_1159_ Hem/Hol	*A*_1506_/*A*_1159_ Lig/Hol
NW-T	1.82	0.56
DW-T	1.12	0.06
NW-L	2.82	0.92
DW-L	1.42	0.09

a The data have been determined for natural wood (NW) and delignified wood (DW) for transversal (T) and longitudinal (L) cut sections.

DW showed a remarkable decrease of the Lig/Hol ratio by about 90% with respect to NW, the result was fully comparable for longitudinal (L) and transversal (T) cut samples. The Hem/Hol ratio of DW also decreased, but to a minor extent; the effect was more pronounced for L-wood samples (about 50%) than for T-wood ones (about 38%).

Raman spectroscopy is a powerful and noninvasive analytical technique for chemical and structural analyses of wood that does not require neither sample preprocessing nor complex experimental equipment. Raman spectroscopy is recognized as a complementary technique of IR spectroscopy.^12,61^ Raman patterns of several pristine hardwood and softwood species have been reported in the literature.^57,62^ Due to the multicomponent nature of wood, the vibrational spectrum is expected to show the contributions of cellulose, hemicellulose, and lignin. According to the literature, due to the comparable nature of chemical bonds within the polysaccharide-based polymers, abundance and crystallinity degree, cellulose overlap, and hidden most of the broad and weak contribution of the amorphous hemicellulose component.^63^ On the other side, the Raman spectra of lignocellulosic materials usually show the distinctive vibrational modes of lignin.^12,64^ Since lignin cannot be isolated without modification, the contribution of lignin is usually obtained by comparing in situ information acquired for untreated and delignified or degraded wood.^57^ However, as expected for a NW excited with a 785 nm laser source (see Section 2.4), pristine Paulownia samples experienced laser-induced fluorescence that gave rise to a strong overwhelming background signal.^62^ The normalized Raman spectrum of delignified samples in the fingerprint region 200–1800 cm^–1^ (Figure 4) showed the characteristic features of wood, including the peak at 375 cm^–1^ **(1)** assigned to symmetric CC bending and ring deformation, at 1096 cm^–1^ **(8)** associated with the C–O–C vibration modes of glycosidic bonds in cellulose and at 1600 cm^–1^ **(14)** due to the breathing mode of the phenyl rings (symmetrical stretching of the aryl rings) in lignin, whose intensity depends on the content of lignin monomers (S, G, or H).^12,57,63–66^ The strong peaks in the low-frequency range 200–700 cm^–1^ (i.e., **(2)** 430 cm^–1^, **(3)** 456 cm^–1^, and (**4**) 520 cm^–1^) reflect the joint contribution of vibrational modes of amorphous and crystalline cellulose domains and hemicellulose.^63^ Moreover, the three bands **(5)** at 797 cm^–1^, **(10)** at 1268 cm^–1^, and **(15)** at 1660 cm^–1^ are markers of guaiacyl lignin subunits (G), whereas the four peaks at 895 cm^–1^ **(6)**, 993 cm^–1^ **(7)**, 1333 cm^–1^ **(11)**, and 1375 cm^–1^ (**12**) are recognized to belong to cellulose vibration modes.^25,57,67^ The overlapped contribution of cellulose and hemicellulose is associated with peak (**9**) 1120 cm^–1^ and that of lignin and cellulose to the vibration mode located at 1461 cm^–1^ **(13)**.^57,67^

**Figure 4:**
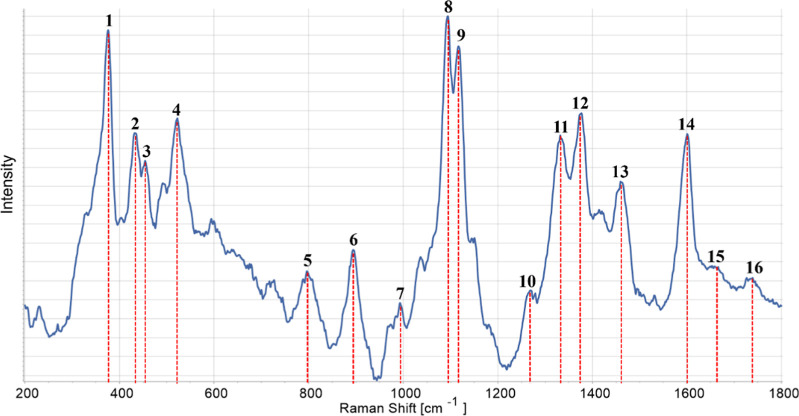
Normalized Raman spectrum of delignified wood (DW). For visualization purposes, the individual spectra were normalized to the highest peak. Numerical annotations are explained in the text.

It can be concluded that the chosen delignification procedure allowed the degradation of lignin chromophores, resulting in bulk samples (DW) characterized by significantly lower fluorescence even if excited with a 785 nm laser source.^12^ In line with this evidence, the spectrum of DW (Figure 4) also showed the weak bands around 1660 cm^–1^ **(15)** and 1735 cm^–1^ **(16)**, possibly contributed to conjugated carbonyl groups formed in the residual lignin upon chemical delignification treatment in acid chlorite.^12,64^ Interestingly, the overall pattern of the Raman spectrum (Figure 5) in the range 850–1800 cm^–1^ is fully comparable with that reported by Agarwal et al.^63^ for Eucalyptus wood, also a hardwood species, delignified in an acidic chlorite medium.

**Figure 5:**
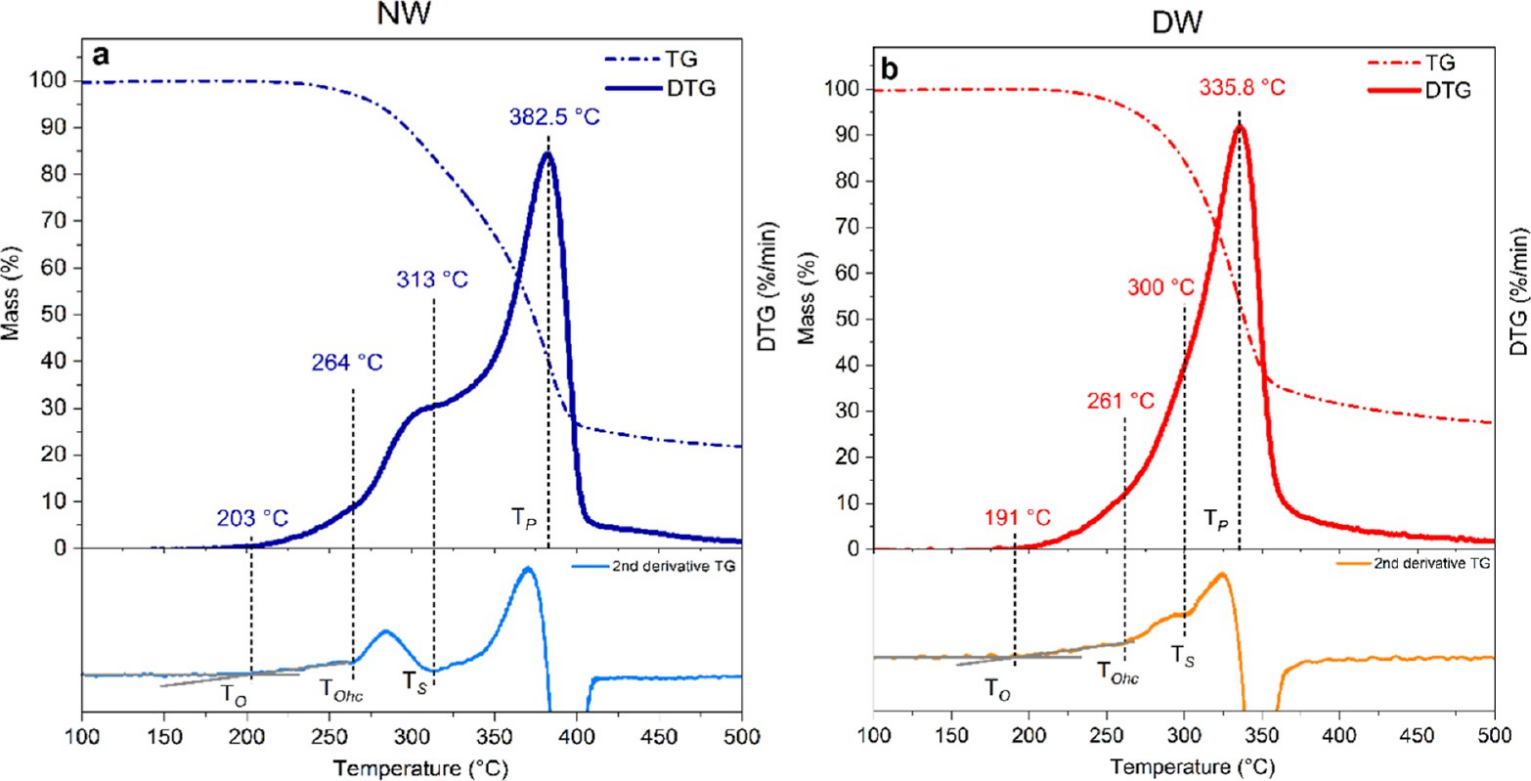
TG, DTG, and second derivative of TG signal for Paulownia natural wood (NW, chart a) and delignified wood (DW, chart b): characteristic temperatures are reported.

The thermal degradation profiles of Paulownia NW and delignified wood (DW) are reported in Figure 5a,b, respectively. Analyzing the first and second derivatives of the TG profile, four characteristic temperatures can be defined:^56,68^ the onset temperature (*T*_O_) that indicates the beginning of wood decomposition; the onset of hemicellulose decomposition (*T*_Ohc_); the temperature of maximum hemicellulose decomposition rate, indicated by a shoulder on the main peak of the DTG profile (*T*_S_); and the temperature of maximum cellulose decomposition rate, corresponding to the DTG main peak (*T*_P_).

It is well-known that the pyrolysis of wood usually occurs above 270 °C, while gasification of wood requires temperatures higher than 500 °C.^7^ The low-temperature event in the pyrolysis of pristine wood (blue curve) is mainly associated with the pyrolysis of hemicellulose whose low thermal stability is associated with the amorphous branched structure and the low molecular weight. The main peak in the DTG pattern mainly concerns the thermal degradation of cellulose, starting with the amorphous domains and progressively involving the more stable crystalline phase.^69,70^ As commonly observed for several other wood species, under the adopted conditions called general pyrolysis, the third step related to the degradation of lignin has not been detected. Due to the complex cross-linked structure and the high molecular weight, lignin undergoes thermal degradation over a wide temperature range (about 150–900 °C) and requires significantly more energy to degrade than hemicellulose and cellulose. The weight loss is associated with the release of CO and CO_2_, prevalently from the polysaccharide components, especially hemicellulose and CH_4_ whose higher yield is mainly associated with the thermal degradation of lignin. Most other organic compounds are released below 500 °C from hemicellulose and cellulose. The composition of lignocellulosic materials substantially contributes to their thermal stability, with higher contents of extractives and hemicelluloses leading to earlier degradation, whereas higher lignin content, resulting in improved thermal stability.^22^ NW thermogravimetric analysis in Figure 5a displays *T*_O_ = 203 °C, *T*_Ohc_ = 264 °C, a clearly visible contribution due to hemicellulose pyrolysis, with *T*_S_ = 313 °C, and cellulose degradation at 382.5 °C. The overall weight loss at 500 °C is 78.2%. The same analysis on DW resulted in slightly lower *T*_O_, *T*_Ohc_, and *T*_S_ values (Figure 5b). The hemicellulose pyrolysis contribution is less distinguishable, as it overlapped with the main peak related to cellulose degradation, which for DW occurs 47 °C earlier than for NW, at 335.8 °C, leading to a final mass loss of 72.5%. As previously observed, the delignification process resulted in a consistent decrease in lignin content (Table 1 and peaks (3) and (4) in Figure 3), also affecting the amount of hemicellulose (Table 1 and peak (9) in Figure 3). This significantly affected the thermal stability of the NW, shifting the main DTG peak to lower temperatures.

XRD is one of the primary techniques used for estimating the crystallinity of cellulosic materials. The Segal method is a simple methodology to estimate the relative changes of cellulose crystallinity in lignocellulosic materials due to the exposure of samples to chemical, physical, or biological treatments.^45,46,63,66^ Figure 6 presents the XRD spectra of NW and delignified wood (DW). Cellulose crystal lattice planes have been designed and assigned according to French.^71^ The calculated Segal crystalline index (CI, %) and crystallite size (τ, nm) are reported in Table 2.

**Figure 6:**
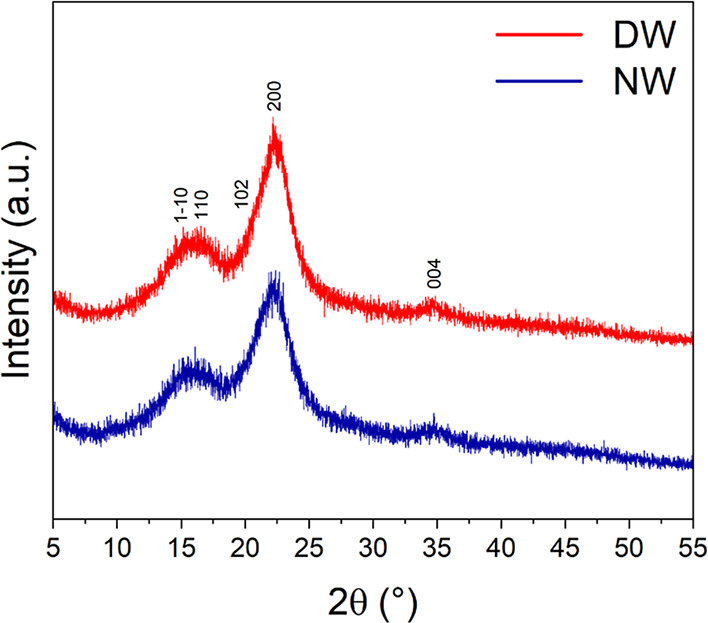
XRD of natural wood (NW) and delignified wood (DW).

**Table 2:** Segal Crystalline Index (%, CI) and Crystallite Size (τ, nm) of Natural Wood (NW) and Delignified Wood (DW)

sample	Segal CI (%)	τ (200) peak (nm)
NW	53.1	2.68 ± 0.01
DW	58.6	2.91 ± 0.02

The results showed that DW is characterized by a slight increase in Segal CI (%) and crystallite size.

It was recently reported^22^ how crystallinity and crystallite size significantly increased (+25% Segal CI and +41% τ, respectively) after a 3 h delignification in alkaline sulfite medium that led to consistent removal of hemicellulose and extractives. In this case, the delignification process caused the breakage of the aromatic skeleton of lignin as well as the reduction of hemicellulose content (Table 1). This resulted in a milder increase in crystallinity (+10% Segal CI) and crystallite size (+9% τ).

Photographs of T-cut and L-cut of NW, control sample, DW, and aesthetic TW are compared in Figure 7. In the case of wood-T samples (Figures 7a and 8), the chosen chemical delignification procedure clearly induced a spatial-selective removal of colored components, giving rise to templates characterized by alternating whitish and yellowish regions corresponding, respectively, to EW and LW regions. The intrusion with the amber colored bioresin allowed enhancement of the transparency and the contrast between EW and LW, favoring the achievement of TW products that preserved the natural pattern of Paulownia wood (Figures 7a and 8).

**Figure 7:**
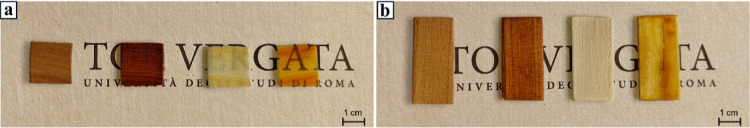
Photographs of (a) T-cut and (b) L-cut of (from left to right): natural wood (NW), control sample (CS), delignified wood (DW), and aesthetic transparent wood (TW) dried in room condition.

**Figure 8:**
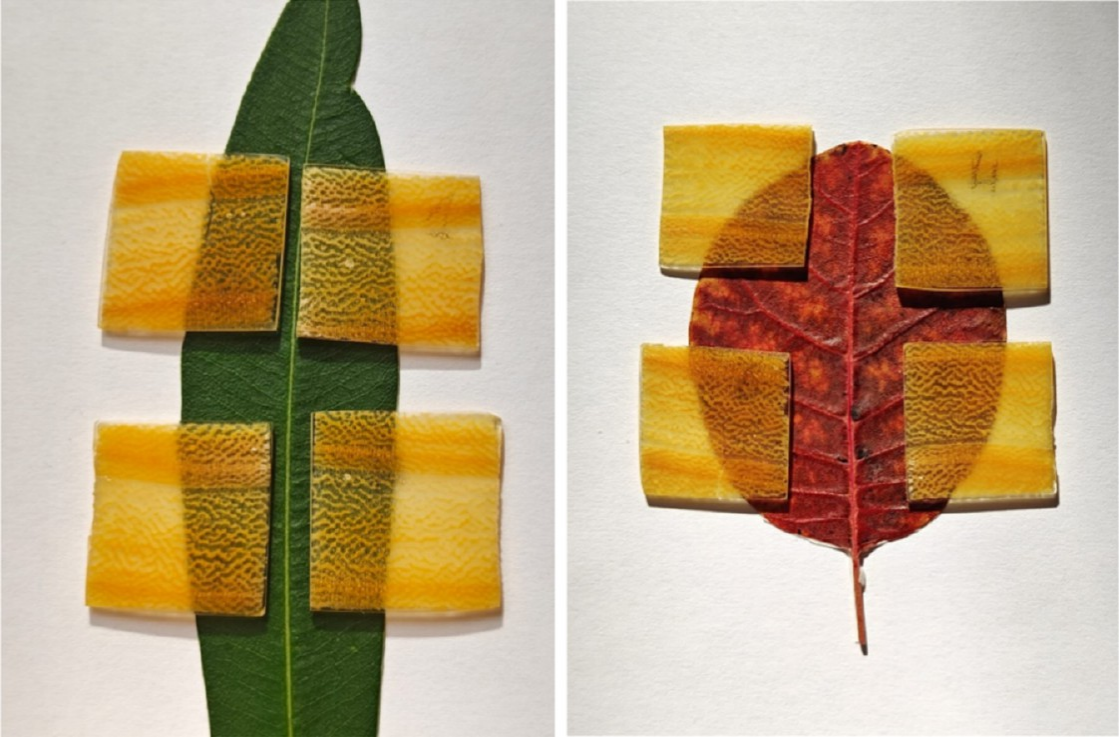
Photographs of aesthetic transparent wood (TW) (transversal cut).

The total transmittance (specular and diffused) of NW and DW in the wavelength range 350–800 nm is reported in Figure 9.

**Figure 9:**
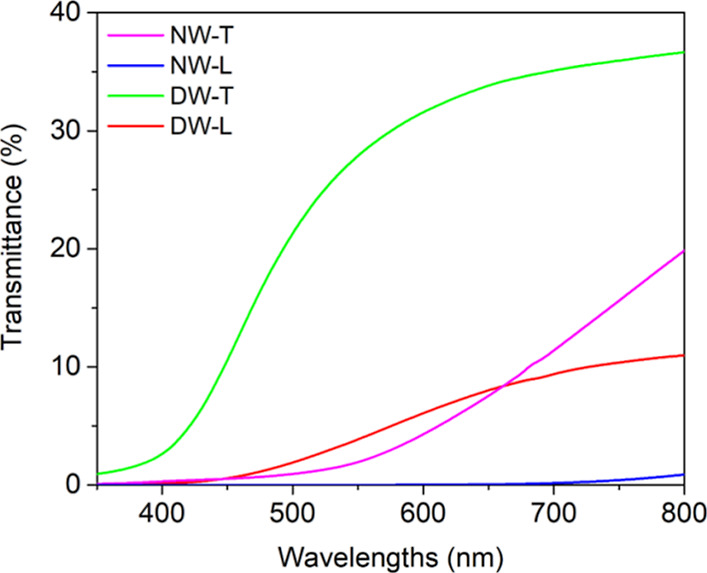
Total transmittance spectra acquired in the UV–vis range 350–800 nm of natural woods (NW) and delignified woods (DW) for transversal (T) and longitudinal (L) cut sections.

Pristine bulk wood showed very low total transmittance, reaching the maximum values at 800 nm of 1% for L-wood and <10% for T-wood.^22,72^ According to the obtained slope of the curve, the transmission in the UV–vis wavelength range is dominated by the absorption of lignin either associated with the aromatic phenylpropane units or to various chromophoric elements, including chromophoric functional groups (i.e., phenolic and hydroxyl groups, double bonds, and carbonyl groups), chromophoric systems (i.e., quinones and biphenyls), leucochromophoric systems (i.e., methylenequinones, phenanthrenequinones, and phenylnaphthalenediones), intermediates (free radicals), and complexes (i.e., chelate structures bound to metal ions).^7,14^ Interestingly, absorption increases by increasing the concentration of absorbing elements that are higher for longitudinal-cut samples than for transversal-cut ones.^73^

In addition to absorption phenomena, the anisotropy of wood also induces significant scattering effects, particularly at shorter wavelengths, where limited transmittance occurs. These effects are especially pronounced in L-cut samples (Figure 2a,c).^54,72^

Chemical delignification led to bulk wood templates characterized by remarkably improved transparency in the investigated range. Despite many authors reporting transmittance at 550 nm, in this work, we adopt 800 nm as the reference wavelength, following the approach established by Wu et al. (2019).^37,74^ At 800 nm, the total transmittance of T-cut samples nearly doubled (i.e., 20% for NW and 38% for DW), whereas for L-cut samples an increase by about ten times (i.e. 1% for NW and 10% for DW) was observed.

As expected, the curves now show only absorption in the UV range. Noticeably, the optical properties of DW maintained the anisotropic character previously observed for NW.^54,72^ Therefore, the improved transparency observed for DW has to be mainly associated with the removal of aromatic components, such as lignin, that are mainly responsible for light absorption in the UV–vis range. Interestingly, the trend of the UV–vis spectrum recorded for the DW-T (Figure 9) shows a small transmission of a few % even at 350 nm, which is an indication of light transmitted through the large pores that cross the sample section.^21,22,54^

The optical properties of TWTW and CS are presented in Figure 10. For the sake of comparison, the results of NW and DW discussed above (Figure 9) have also been included in Figure 10. The infusion of delignified bulk wood samples (DW) with the epoxy bioresin led to materials (TW) characterized by remarkably improved transparency, i.e., about +65% and +82% at 800 nm for T-cut and L-cut, respectively, compared to the corresponding T-DW and L-DW samples. The highest *T* % value was obtained for aesthetic transversal-cut TW (TW-T) that reached 61% at 800 nm (Figure 10a), putting into evidence that the delignification treatment greatly favored the infiltration of the epoxy bioresin within the hollow delignified anatomical elements.

**Figure 10:**
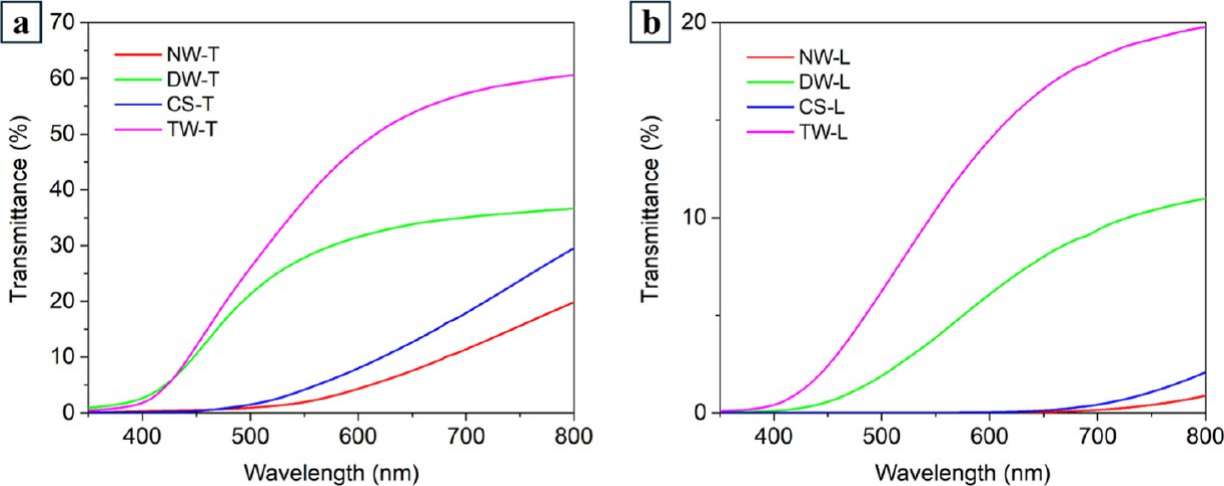
Total transmittance curves acquired in the UV–vis range 350–800 nm for: (a) T-cut and (b) L-cut natural wood (NW), delignified wood (DW), aesthetic transparent wood (TW), and control sample (CS).

The enhanced light transmittance is clearly due to the significant reduction in light scattering resulting from the good refractive index matching between the wood structure and the polymer matrix.

This is confirmed by what is reported in the literature,^23,38^ showing that TW products are obtained using refractive index-matched polymers, mainly poly­(methyl methacrylate) (PMMA) (*n* ≈ 1.49) and ER (*n* ≈ 1.5).^54^

In this study, the aesthetic TW derived from 1 mm-thick transversal cut samples and longitudinal cut of Paulownia reached a total transmittance, respectively, of 60% at 800 nm (50% at 600 nm) and 20% at 800 nm (15% at 600 nm). On a wide basis, besides the content of residual lignin, the optical transmittance (*T* %) of TW ranges within 6% and 98%, depending on multiple concurrent factor including reference wavelengths, delignification process parameters, wood species and cut, orientation of cellulose fibers, cellulose volume fraction, sample thickness, and wood-polymer interface.^15,29,54,75^ Actually, most of TW products are characterized by an optical transmittance that varies approximately between 60% and 85%, only a minority show a transparency higher than 90% or lower than 60%.^15,29,75^

Regarding explicitly “aesthetic TW” products, only a few papers are available in the literature. For example, Mi et al. (2020)^25^ reported a total transmittance of 80% at 600 nm for 2 mm-thick aesthetic TW derived from transversal-cut samples of Douglas Fir, whereas Zhou and Xu (2023)^76^ obtained 1 mm-thick aesthetic TW from longitudinal-cut Fir characterized by 50% total transmittance of at 550 nm.

In this whole framework, the obtained optical transmittance values of samples derived from T-cut Paulownia wood (i.e., 60% at 800 nm and 50% at 600 nm) can be related to the specific wood species and to the chosen time-efficient lignin-retaining chemical treatment (paragraph 1, paragraph 2.2) as well as, reasonably, to the presence of an uneven wood-polymer interface (see the following section, Figure 12).

The color spectrophotometer allowed for the determination of the brightness *L* and the chromatic coordinates *a* and *b* of NW, DW, aesthetic TWs, and CS (Figure 11).

**Figure 11:**
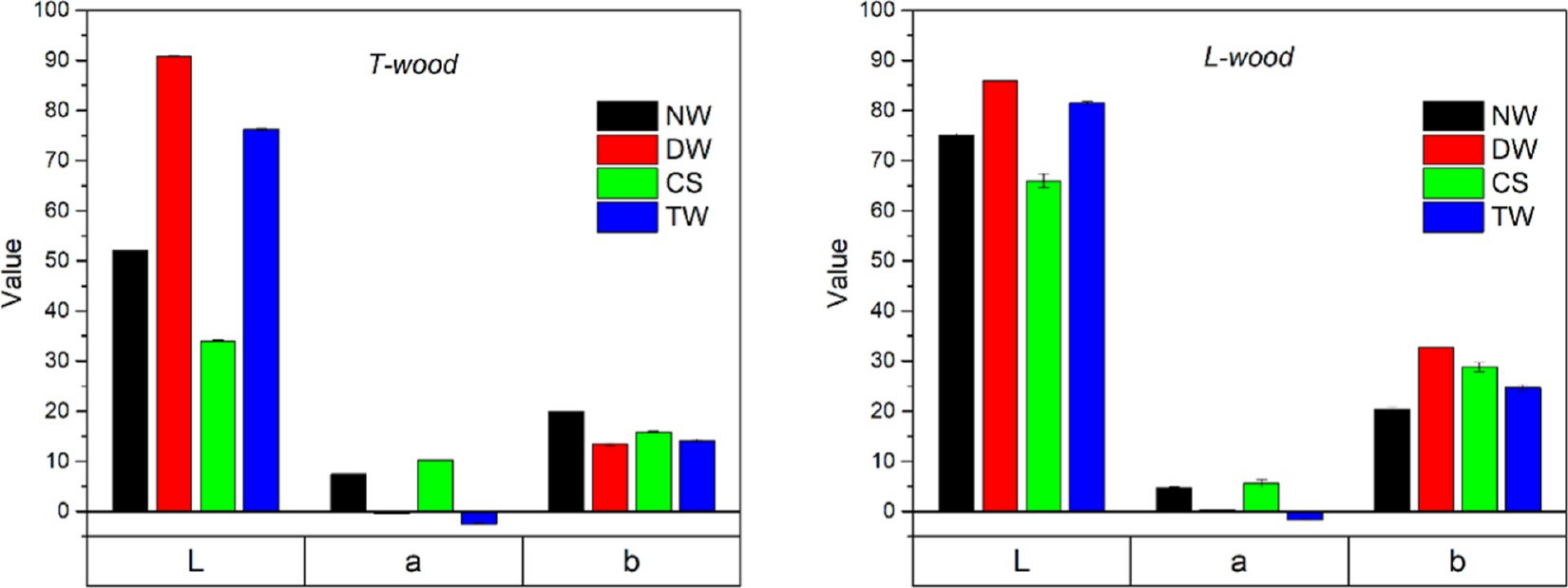
Brightness (*L*) and chromatic coordinates (*a*, *b*) of natural wood (NW), delignified wood (DW), aesthetic transparent wood (TW), and control sample (CS) on T-wood (left) and L-wood (right).

The coordinates of CS in the CIELAB system are (34, 10, 16) and (66, 6, 29) for T-wood and L-wood, respectively. The result is in line with the values reported in the literature for colored hardwood species.^49^ It is worth mentioning that a certain degree of variability has to be expected since the measurement is sensitive to the state of the surface and to the cut section (i.e. transversal and radial longitudinal)^49^ (Figures 1 and 2a,c). The coordinates of the TWs in the CIE LAB system are (76, −2, 14) and (81, −2, 25) for the T-wood and L-wood, respectively. The change of color Δ*E* due to the delignification treatment of Paulownia wood (couples #1) is 40.1 and 17 for T-wood and L-wood, respectively (Table S1, Supporting Information). According to a scale originally proposed by Minemura and Umehara,^77^ the eye can fully appreciate a change of color when the Δ*E* value reaches at least 12.^49^ For brightness (Δ*L*), Phelps and Mc Ginnes^78^ estimated that a variation of minimum 3% is required. On this basis, the change of color and brightness of natural Paulownia wood after delignification is also evident in photographs reported in Figure 7 (Table S1, Supporting Information).

L strongly increases in the delignified samples (DW and TW), with a double value with respect to the original one because of the removal of absorbing moieties with the treatment, and this is apparent from the photo in Figure 7. Moreover, it can be noticed that CS shows lower brightness than NW because the resin increases the amount of light passing through the wood, thereby reducing the reflected light and consequently decreasing the *L* value. In addition, the ER is colored, thus contributing to the absorption and reducing the brightness. Focusing on *a* parameter (Figure 11), it drops to very low values for delignified wood.^26^ As for the *b* value, we do not see important variations between the samples; this is not surprising because the color in the wood samples is dominated by the red component of lignin-based chromophores. In conclusion, the apparent change of color upon delignification is driven by the change of brightness.

The micrographs of aesthetic TW obtained by infiltration of the epoxy bioresin within transversally cut delignified wood are reported in Figure 12. A complete infiltration was achieved, and the framework of the original hardwood microstructure was maintained (Figure 2). Accordingly, the fabrication of TW-T samples was accompanied by a weight gain of 460 ± 50% that was much higher than that recorded for the control (i.e., 260 ± 50%). Therefore, a strong interaction is expected between the cellulose-rich template and the polymeric phase.^40,72^

**Figure 12:**
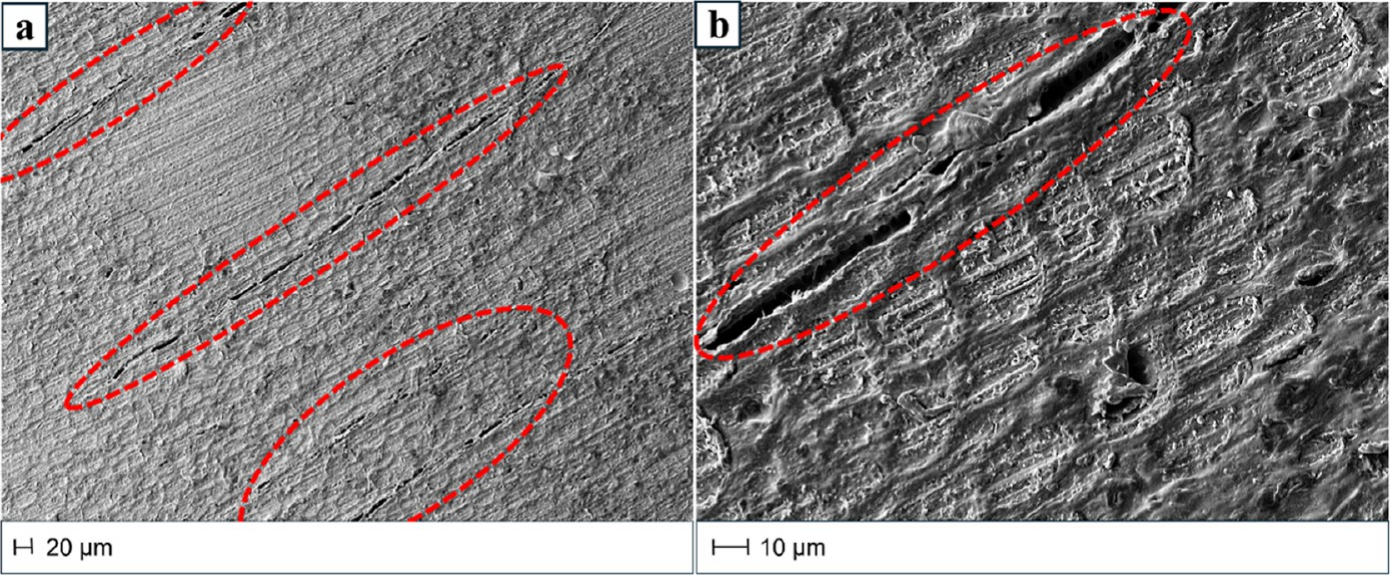
Scanning electron microscopy (SEM) micrographs of T-cut aesthetic transparent wood (TW-T) from lower (a) to higher (b) magnification. The dotted red lines indicate some defects.

In Figure 13, the stress–strain curves representative of the average performance for the tested NW, delignified wood, and TW specimens are reported. Tensile tests show that the bleaching treatment led to delignified templates (DW) characterized by decreased tensile strength (σ_TS_) with respect to pristine wood (i.e., 21 ± 9 MPa and 41 ± 12 MPa, *p* = 0.015). The mechanical properties of Paulownia were fully recovered (i.e., 41 ± 12 MPa and 34 ± 9 MPa, *p* = 0.23) after the intrusion with the epoxy bioresin (TW) (Figure 13), evidencing that cellulose microfibers maintained their pristine orientation and that good interaction occurred at the interface with the epoxy bioresin due to cross-linking between the delignified cellulose-rich scaffold and the ER.^54,72^

**Figure 13:**
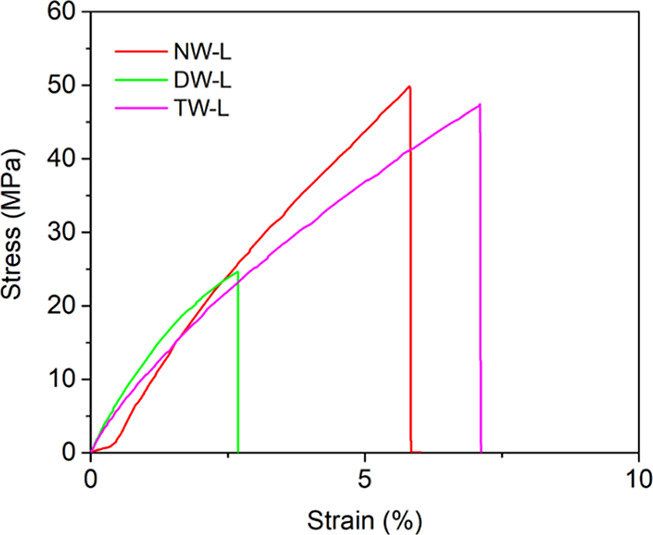
Stress–strain curve of longitudinal-cut (L) samples: pristine wood (NW), delignified wood (DW), and aesthetic transparent wood (TW).

However, reasonably, the presence of unpaired wood-polymer interface zones and/or filling defectiveness, as suggested by the details evidenced in Figure 12, is responsible for the lack of the expected reinforcement effect (paragraph 2.3).

Finally, it is worth mentioning that, on the basis of the most recent reviews,^15,29,75^ a number of papers on TW do not report data on the mechanical properties of the obtained products. Moreover, the available data cover a wide range of different combinations in terms of wood species, cut, delignification route, process parameters, and filled polymer. Restricting to any wood template infused with ER, the values of tensile strength reported in the literature range approximately within 20 and 90 MPa, the great majority falling within the range 20–45 MPa.^15,29,75^ On this basis, it is possible to conclude that the obtained σ_TS_ values are in good agreement with the literature.

Finally, to demonstrate the innovative contribution of this work within the field and put into evidence core advantages and limitations, a detailed comparative analysis in terms of raw materials, preparation methods, and key performance metrics is reported in Table 3.

**Table 3:** Direct Comparisons across Multiple Sizes of Transparent Wood Products Obtained from Delignified Wood Templates Infused with Epoxy Resin

wood thickness	delignification procedure	transmittance	tensile strength (MPa)	epoxy resin	ref
Paulownia	NaClO_2_, 3 h	60%@800 nm T-cut	no data (T-cut)	biobased	this study
1 mm T-cut		20%@800 nm L-cut	34 ± 9 (L-cut)		
1 mm L-cut					
Basswood	NaOH + Na_2_SO_3_, 7 h	90%@800 nm T-cut	23 (T-cut)	fossil-based	^29,72,75^
3 mm T-cut	H_2_O_2_, 5 h	80%@800 nm L-cut	45 (L-cut)		
3 mm L-cut					
Basswood	H_2_O_2_, 4 h	90%@780 nm T-cut	no data (T-cut)	fossil-based	^29,79^
0.8 mm T-cut	H_2_O_2_, 4–12 h	40–87%@550 nm L-cut	21 (L-cut)		
5–20 mm L-cut					
Balsa	NaOH + H_2_O_2_/UV, 1 h	90%@400–800 nm T-cut and L-cut	31 (T-cut)	fossil-based	^29,60^
0.6–1.5 mm L-cut			46 (L-cut)		
1–3.3 mm T-cut					
Douglas Fir	NaClO_2_, 2 h	>80%@600 nm T-cut	22 (T-cut)	fossil-based	^25,75^
0.6–2 mm T-cut, L-cut			92 (L-cut)		
Basswood	NaClO_2_, 2 h, NaClO, 72 h	85–90%@550 nm L-cut	33 (T-cut)	fossil-based	^19,75^
0.7–1.5 mm L-cut			44 (L-cut)		
Balsa	NaClO_2_, 1–6 h	10–80% L-cut (no data on reference wavelength)	4–63 (T-cut)	fossil-based	^75,80^
1–5 mm L-cut			45–75 (L-cut)		
Basswood	NaOH + Na_2_SO_3_, 3 h, H_2_O_2_, 2–3 h	>80% T-cut (no data on reference wavelength)	11.7 (T-cut)	fossil-based	^15,81^
0.3–1.4 cm T-cut					
Balsa	NaOH + Na_2_SO_3_	80%@550 nm L-cut	no data	fossil-based	^15,82^
1 mm L-cut					

## 4 Conclusions

*Paulownia tomentosa* is a cultivation fast-growing hardwood species characterized by a semi-ring porous structure with large vessels (∼100 μm) mainly located in the EW region.

Aesthetic TW products were obtained from 1 mm-thick Paulownia wood-T (transversal cut) and wood-L (longitudinal-cut) samples. The chemical delignification was efficiently and rapidly performed following a simple bleaching route based on an acid sodium chlorite solution. The recorded dry mass loss (10 to 15% by weight) was mainly due to the removal of lignin and, to a minor extent especially in the case of wood-T samples, to the removal of hemicellulose. Accordingly, delignified templates showed increased average transparency and brightness accompanied by loss of thermal stability, increased Segal CI and crystallite size, and decay of tensile strength, mainly correlated to the degradation of the aromatic lignin network.

The delignified wood-T and wood-L templates were then vacuum impregnated with a biologically derived ER. The obtained wood-polymer hybrids fully recovered the tensile properties (about 40 MPa) and showed a remarkable gain in light transmittance. The biobased composite derived from wood-T samples reached 60% of total transmittance (at 800 nm) and, simultaneously, displayed attractive aesthetic features.

Concluding, lignin has been intentionally retained to preserve the natural colors and patterns of Paulownia wood to secure the typical chromatic features expected for an aesthetic wood product. The moderate optical and mechanical properties of the obtained bioderived composites make them mainly devoted to applications in design, green building, and architecture.

It is worth mentioning that the performance of the obtained aesthetic TW is influenced by a number of key factors, including the degree of delignification, the amount of loaded epoxy bioresin, the quality of wood-polymer interphase, and the thickness of bulk wood samples, which will be addressed in further studies.

Finally, future developments of TW products are expected to be directed toward the reduction of the carbon footprint by either exploring alternative delignification routes and/or employing biodegradable, environmentally friendly polymers, and toward the investigation of durability in an outdoor environment.

## Supplementary Material



## Notes

Bioresin transmittance, SEM control sample, and brightness variation table (PDF)
