# Supplementary material for: Toward Sustainable Aesthetic Transparent Wood from a Fast-Growing Hardwood Species: Paulownia Wood Templates Infused with Epoxy Bioresin
Source: ACS Omega. 2026 Jan 8;11(2):3031–45. doi: 10.1021/acsomega.5c09133 (PMC12824965; doi:10.1021/acsomega.5c09133)
Supplement: Supplementary file 1 [file ao5c09133_si_001.pdf]

SUPPLEMENTARY MATERIAL OF THE MANUSCRIPT

Toward sustainable aesthetic transparent wood from a fast-growing hardwood species: Paulownia wood templates infused with epoxy bioresin

Francesco Bolognesi [a,e], Emanuele Galvanetto [b], Leonardo Duranti [c], Andrea Bianco [d], Marco Togni [e] and Alessandra Bianco [a]*

(a) Università degli Studi di Roma "Tor Vergata", Dipartimento Ingegneria dell'Impresa "Mario Lucertini", Consortium INSTM RU "Roma Tor Vergata", Via del Politecnico, 00133 Roma, Italy.

(b) Università degli Studi di Firenze, Dipartimento di Ingegneria Industriale (DIEF), Via di Santa Marta 3, 50139 Firenze, Italy.

(c) Università degli Studi di Roma "Tor Vergata", Dipartimento di Scienze e Tecnologie Chimiche, Consortium INSTM RU "Roma Tor Vergata", Via della Ricerca Scientifica, 00133 Roma, Italy.

(d) INAF Osservatorio Astronomico di Brera, via Bianchi 46, 23807 Merate, Italy.

(e) Università degli Studi di Firenze, Dipartimento di Scienze e Tecnologie Agrarie, Alimentari, Ambientali e Forestali (DAGRI), Piazzale delle Cascine 18, 50144 Firenze, Italy.

Corresponding author

Supporting information and Supplementary material

Contents: bioresin transmittance (Fig. S1); SEM control sample (Fig. S2); brightness variation table (Table S1)

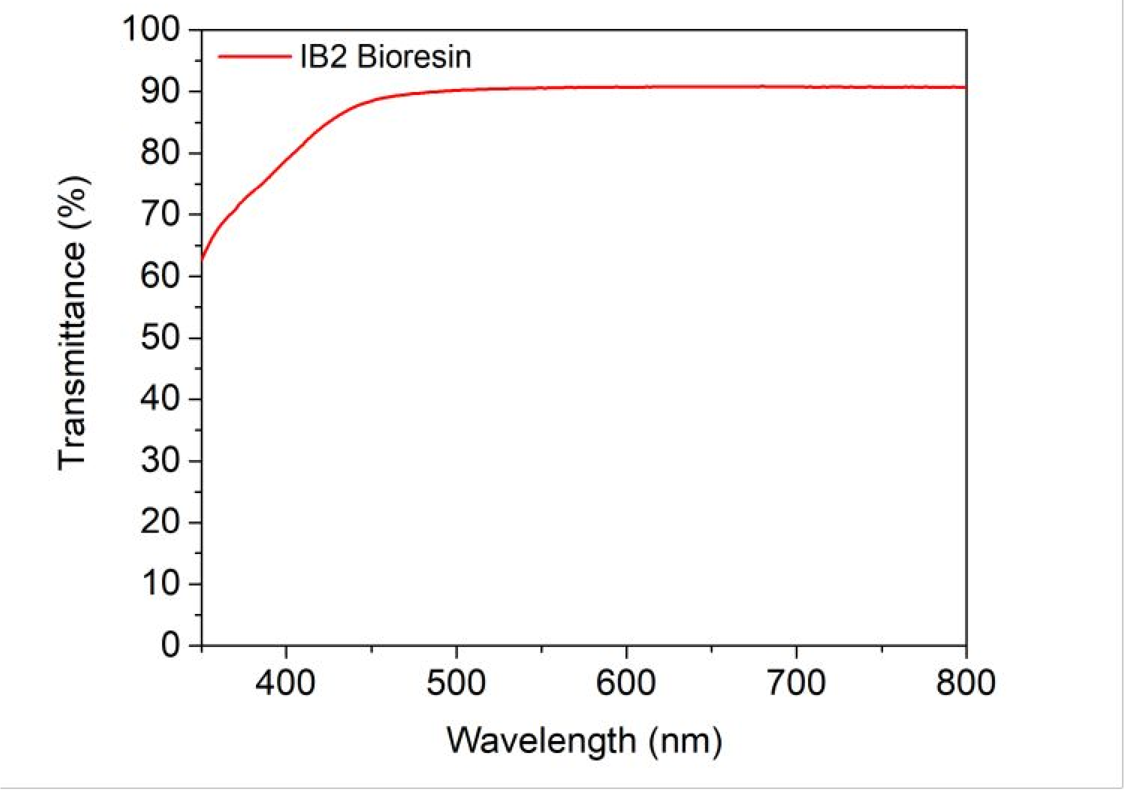

Fig. S1
Transmittance (T%) of the epoxy bioresin

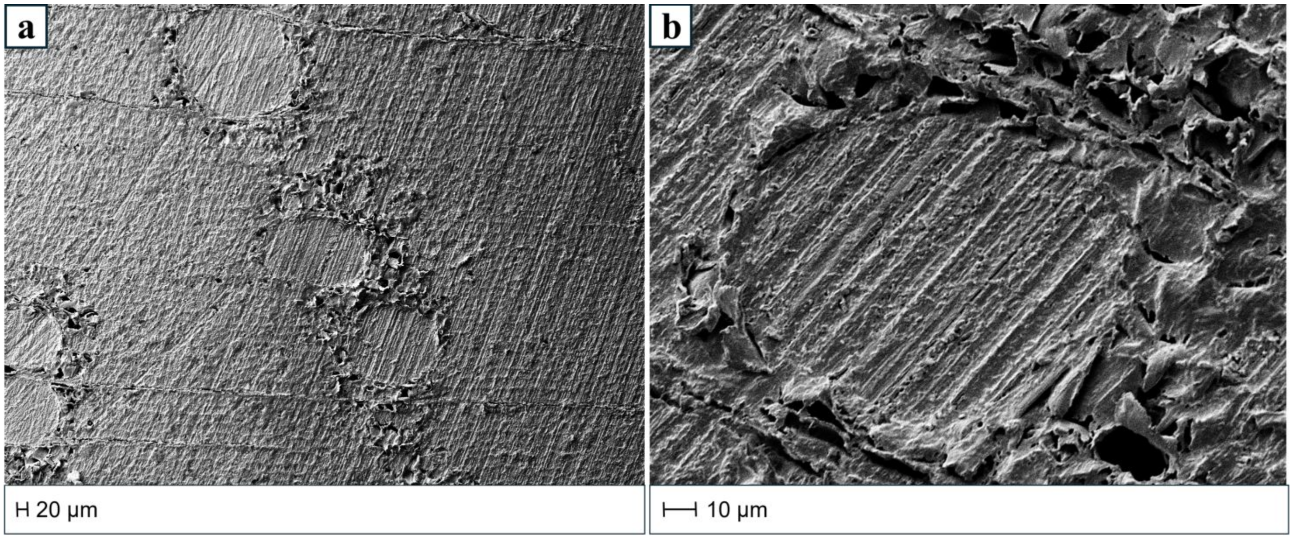

Fig. S2

Scanning Electron microscopy of control sample (CS)

Table S1

Brightness variation (*ΔL)* and colour change (*ΔE)*

Couples #1	*ΔE**<br>*ΔL***	Couples #2	*ΔE**<br>*ΔL***	Couples #3	*ΔE**<br>*ΔL***
NW-DW (T-wood)	40.1*<br>+39**	NW-TW (T-wood)	26.7*<br>+24**	CS-TW (T-wood)	44.1*<br>+42**
NW-DW (L-wood)	17*<br>+11**	NW-TW (L-wood)	10.1*<br>+6**	CS-TW (L-wood)	17.6*<br>+15**
